# Barriers and facilitators to implementation of menu labelling interventions to support healthy food choices: a mixed methods systematic review protocol

**DOI:** 10.1186/s13643-018-0752-3

**Published:** 2018-06-23

**Authors:** Claire Kerins, Jennifer McSharry, Catherine Hayes, Ivan J. Perry, Fiona Geaney, Colette Kelly

**Affiliations:** 1 Discipline of Health Promotion, School of Health Sciences, National University of Ireland Galway, University Road, Galway, Ireland; 2 Health Behaviour Change Research Group, School of Psychology, National University of Ireland Galway, University Road, Galway, Ireland; 3 Discipline of Public Health and Primary Care, Institute of Population Health, Trinity College Dublin Russell Centre, Tallaght Cross, Dublin 24, Ireland; 4 School of Public Health, University College Cork, College Road, Cork, Ireland

**Keywords:** Menu labelling, Obesity, Implementation, Barriers, Facilitators, Mixed methods, Systematic review, Consolidated Framework for Implementation Research, Best fit framework synthesis

## Abstract

**Background:**

Menu labelling is continuing to gather public and legislative support as one of the potential environmental strategies for addressing the obesity pandemic. However, issues relating to implementation have been reported in countries where menu labelling has been introduced on a voluntary or mandatory basis. The aim of this mixed methods systematic review is to synthesise the empirical evidence on the barriers and facilitators to implementation of menu labelling interventions to support healthy food choices.

**Methods:**

This review will use the ‘best fit’ framework synthesis approach to synthesise qualitative, quantitative and mixed methods evidence. Peer-reviewed publications will be accessed through PubMed, EMBASE, CINAHL, PsycINFO, Web of Science and Scopus. Grey literature will be accessed through Google Scholar, OpenGrey, RIAN, EThOS, ProQuest, WorldCat, Networked Digital Library of Theses and Dissertations, Open Access Theses and Dissertations, and public health organisation websites. Screening reference lists, citation chaining and contacting authors of all included studies will be undertaken. There will be no restriction on menu labelling scheme or format, publication year or language; however, only primary research studies relevant to supply-side stakeholders will be eligible for inclusion. Study quality will be assessed using the Mixed Methods Appraisal Tool. At least two independent reviewers will perform study selection, data extraction and quality appraisal; if consensus is required, another independent reviewer will be consulted. A combination of deductive coding, using the Consolidated Framework for Implementation Research as the a priori framework, and inductive analysis, using secondary thematic analysis, will be used. The overall process will assist in the construction of a new evidence-based conceptual model regarding the implementation of menu labelling interventions. The new model will be assessed for bias and a sensitivity analysis performed.

**Discussion:**

Given the growing consensus that a systemic, sustained portfolio of obesity prevention strategies, delivered at scale, is needed to address the obesity epidemic, greater understanding of the practical issues relating to implementation of such strategies is required. Findings from this review will be used to develop a set of best-practice guidelines to enhance the adoption, implementation and sustainability of menu labelling interventions across countries worldwide.

**Systematic review registration:**

PROSPERO CRD42017083306

**Electronic supplementary material:**

The online version of this article (10.1186/s13643-018-0752-3) contains supplementary material, which is available to authorized users.

## Background

The prevalence of overweight and obesity is increasing worldwide [1]. This represents a major public health challenge due to the associated morbidity and mortality from diet-related diseases such as type 2 diabetes, cardiovascular disease and cancer. In response to this, a number of public health strategies have been developed to improve dietary patterns at a population level. One such strategy includes menu labelling which aims to improve the availability and visibility of healthy foods when eating outside the home; therefore, making the healthy choice the easy choice [2, 3]. Menu labelling applies food labelling principles to the out-of-home eating environment through the provision of nutrition information on menus at the point of sale.

While no single intervention will address the obesity epidemic, menu labelling can form part of a systemic, sustained portfolio of environmental interventions, implemented on a large scale, in order to address this epidemic [4]. To date, a number of countries and regions around the world have introduced menu labelling on a mandatory basis. These include the United States of America (USA) [5], Australia [6] and more recently the Province of Ontario in Canada [7]. Other countries, including the United Kingdom (UK) [8] and the Republic of Ireland (ROI) [9], have implemented voluntary menu labelling schemes.

Similarly, a number of workplace and healthcare organisations have independently developed and implemented menu labelling policies at national and local levels [10–12]. These policies have been developed in recognition of the role of both workplace and healthcare settings in promoting healthy behaviours [13, 14]. The healthcare setting, with responsibilities to both employees and patients, now increasingly recognise their leadership position in serving as public health role models as well as health promotion advocates [15]. In developing and implementing menu labelling policies, a wide range of supply-side stakeholders have been involved including researchers, healthcare professionals, policy-makers, caterers, food service business staff and management, and restaurant/foodservice associations.

In general, menu labelling has gathered growing public and legislative support in response to the increased consumption of foods prepared outside the home and the associated risks of overweight and obesity [16]. Furthermore, demand-side stakeholders (i.e. consumers) now seek more transparency in the nutritional value of food consumed outside the home [17, 18]. In the ROI, it is estimated that adults consume 24% of their total energy from food and drink outside the home [19]. Similarly, in the USA, adults consume on average 11.3% of their energy intake from fast-food [20]. Furthermore, it is estimated that working adults consume at least one meal per day in workplace canteens [21]. Research has shown that eating outside the home is associated with higher energy and fat intake, lower micronutrients intake [22, 23], increased body fat [24] and weight gain [16]. Moreover, research demonstrates that adults underestimate the calorie, fat and sodium content of menu items when eating outside the home [25, 26].

A number of systematic reviews have examined the effectiveness of menu labelling on consumer food choice [27–29]. While the results of these have been mixed, new evidence from a Cochrane review suggests calorie menu labelling may reduce energy purchased in restaurants [30]. The authors performed a meta-analysis of three randomised controlled trials and found a statistically significant reduction of 47 calories in energy purchased associated with calorie menu labelling [30]. Thus, for an average meal of 600 calories, the size of this effect suggests that calorie menu labelling may reduce energy purchased per meal by 7.8% [30]. However, the authors note the review findings are based on lower-quality studies and highlight the need for further well-conducted studies, in order to establish the size of effect with more precision [30]. One such study includes an adequately powered, properly designed, natural experiment, which found calorie menu labelling was associated with a statistically significant reduction in BMI by 0.38 units [31]. Despite some mixed findings, there is growing consensus that obesity prevention strategies, such as menu labelling, may only have a small effect on individuals but can drive large changes when aggregated at population level [4].

Other impacts of menu labelling include menu reformulation by the food service industry and/or development of new menu items to improve nutritional value. For example, one study found that the calorie content of menu items in a chain restaurant decreased in Washington, following the introduction of mandatory calorie menu labelling [32]. Similarly, an examination of menu calorie content in 44 large chain restaurants before and after the introduction of calorie menu labelling legislation, found a small but statistically significant decline in the mean calorie content of menu items following the introduction of calorie menu labelling [33]. These findings suggest that menu labelling may encourage the food service industry to create healthier food options, thus creating a supportive environment for healthy eating.

Although public health strategies, such as menu labelling, have been developed to improve dietary patterns, it is important to recognise any potential risks associated with such strategies. For example, a recent study reported menu labelling may exacerbate disordered eating tendencies among individuals with eating disorders [34]. However, another study reported no adverse outcomes for individuals at high risk of eating disorders, including those with high levels of disordered eating tendencies [35]. Overall, further research is needed to understand the mechanisms through which menu labelling may impact disordered eating and thus, inform strategies to address these risks.

With more countries implementing voluntary or mandatory menu labelling schemes, issues relating to implementation have arisen. For example, in 2012, the Food Safety Authority of Ireland (FSAI) carried out a national consultation which revealed that one of the main concerns of Irish food service businesses revolved around their lack of expertise in calculating the calorie content of menu items [9]. In response to these concerns, the FSAI launched ‘MenuCal’ in 2014, an online calorie calculator available free-of-charge to food service businesses [36]. However, despite this new resource, a recent evaluation of the uptake of voluntary calorie menu labelling in Ireland revealed a poor level of uptake, with 7% of food service businesses (i.e. fast food chains, cafes, restaurants, pubs) claiming to display calories [37]. The main reasons cited by businesses for not displaying calories included time and cost constraints, and the need for training and support.

Similarly, Thomas [38] refers to the many challenges that the food service industry faces in implementing menu labelling. These barriers range from difficulties in providing accurate nutrition information to the loss of flexibility in changing menu items [38]. In addition, Eyler and colleagues [39] highlight the need for increasing evidence on the benefits of menu labelling for obesity prevention efforts, as well as the need for developing consistent implementation strategies; both of which may facilitate a cultural shift and legislative support. Likewise, Morestin and colleagues [40] discuss the importance of cooperation from the food service industry, as well as the need for careful policy formulation to secure industry support and buy-in. Tied to this, McGuffin et al. [41] stated that for menu labelling policy to be successful, policy-makers must help relevant actors, such as caterers, overcome real and apparent obstacles to implementation.

To date, most systematic reviews have been concerned with determining the effectiveness of menu labelling [27–29]. Thus far, no synthesis of existing research on barriers and facilitators to implementing menu labelling interventions has been conducted. Given the growing consensus that a portfolio of obesity prevention strategies is needed to address the obesity epidemic, greater understanding of the practical issues relating to implementation of such strategies is required. With more countries introducing menu labelling interventions, including workplace and healthcare organisations, lessons learned in terms of implementation can assist others in following suit. The current review protocol seeks to address this gap, by proposing the following ‘policy urgent’ review question: what are stakeholder reported barriers and facilitators to implementing menu labelling interventions to support healthy food choices? Findings from this review will help enhance the adoption, implementation and sustainability of menu labelling interventions across countries world-wide.

The current review will adopt the recently developed ‘best fit’ framework synthesis approach [42]. The ‘best fit’ framework approach is a pragmatic methodology for research synthesis, using a mixture of both deductive and inductive analysis techniques. It offers a means to test, reinforce and build upon existing published conceptual models or frameworks. Furthermore, this approach produces a relatively rapid evidence synthesis and is thus suited to a range of policy urgent questions.

## Review objectives

The primary objective is to identify, appraise and synthesise the existing evidence on barriers and facilitators to implementing menu labelling interventions to support healthy food choices. A secondary objective is to assess the relevance of the identified barriers and facilitators to the workplace setting, and in particular the health care setting.

## Methods

This mixed methods systematic review is registered with the International Prospective Register of Systematic Reviews (PROSPERO): CRD42017083306. The Preferred Reporting Items for Systematic Reviews and Meta-Analysis Protocols (PRISMA-P) checklist has been used in the preparation of this protocol (see Additional file 1) [43]. The review will follow the steps of the ‘best fit’ framework synthesis approach [42] and will be reported following the PRISMA guidelines [44].

### Identification of a pre-existing framework

In this review, the Consolidated Framework for Implementation Research (CFIR) has been chosen as the a priori framework because it was developed following a comprehensive review of the implementation science literature [45]; thus, completing the initial step in the ‘best fit’ framework synthesis approach [42]. This meta-theoretical framework incorporates constructs from existing implementation theories into a single comprehensive framework which can help guide systematic evaluation of potential barriers and facilitators to successful implementation [45]. The CFIR will be used for initial coding of data and will then be updated in response to the emerging synthesis, thus creating a new evidence-based conceptual model regarding the implementation of menu labelling interventions to support healthy food choices.

### Study eligibility criteria

The criteria for study eligibility in this review, including the PICOS acronym (Population, Intervention, Comparison, Outcome, and Study design), are described below.

#### Population

The sample or population of interest is supply-side stakeholders with a role in implementation of menu labelling interventions in food service establishments. This refers to food service business staff and management, caterers, policy-makers, guideline developers, researchers etc. As this review will focus on stakeholders with a direct role in implementation of menu labelling interventions, studies relating to demand-side stakeholders (i.e. consumers) will be excluded, unless data specific to supply-side stakeholders can be extracted separately.

#### Intervention

The intervention consists of menu labelling in food service establishments implemented on a voluntary or mandatory basis. This includes the provision of nutrition information on all or some menu items at the point of purchase. There will be no restriction on menu labelling formats, for example, quantitative formats (e.g. calories, sodium, fat) and qualitative formats (e.g. traffic light labelling, healthy-food symbols) will be included. Studies where menu labelling forms part of a multi-component intervention will be excluded. This decision is based on the anticipated challenges in isolating barriers and facilitators to implementation of menu labelling interventions which form part of a multi-component intervention. All types of food service establishments will be included in this review (e.g. fast food outlets, restaurants, coffee shops, canteens, vending machines).

#### Control

While no comparator is being studied in this review, studies will not be excluded on the basis of having a comparator or control group.

#### Outcome

The primary outcome will include any barrier or facilitator to the implementation of menu labelling interventions to support healthy food choices. A barrier is defined as any variable that impedes or obstructs the implementation of menu labelling. A facilitator is defined as any variable that eases and promotes the implementation of menu labelling. The ‘findings unit’ will include the following: (i) participant quotations from studies using qualitative data collection methods such as interviews and focus groups; (ii) excerpts, quotations or entire passages from studies using the qualitative research method of documentary analysis; (iii) narrative descriptive summaries of results from studies using qualitative data collection methods; and (iv) statistical analyses from studies using quantitative data collection methods such as surveys and questionnaires. The relevance of the identified barriers and facilitators to the implementation of menu labelling interventions in the workplace and healthcare setting will be the secondary outcomes.

#### Study design

The review will include all primary research studies meeting the eligibility criteria. This may include (i) qualitative studies which use appropriate methods of data collection and data analysis (such as case studies, phenomenology, grounded theory, ethnography and action research studies); (ii) quantitative studies (such as cross-sectional studies, case-control studies, cohort studies, quasi-experimental studies, and randomised controlled trials); and (iii) mixed methods studies combining qualitative and quantitative methods of data collection and analysis. Such studies will include, but are not limited to, the following data collection methods: interviews, focus groups, observations, documentary analysis, surveys, and questionnaires. The review will exclude editorials, commentary and opinion pieces; however, these will be used to find further studies.

#### Language

There will be no restriction on language.

#### Publication year

There will be no restriction on publication year.

### Search strategy

The search strategy will be informed by those used in existing reviews of menu labelling and further refined in collaboration with a university librarian. The search strategy will include database-specific controlled vocabularies, free-text words, synonyms, spelling variants and truncation. In order to conduct a comprehensive search, with greater sensitivity than specificity, broad search terms will be used to capture potentially eligible studies. Table 1 includes an example of the proposed search strategy for the PubMed database. The lead review author (CK) will implement the final search strategy when piloted and refined. The following electronic databases will be searched: PubMed, EMBASE, CINAHL, PsycINFO, Web of Science and Scopus. In order to capture relevant information from sources outside the peer-reviewed literature, the review will include grey literature in the search strategy. The types of grey literature will include government or non-governmental organisation reports, research reports, conference proceedings and abstracts, and theses and dissertations. Sources of grey literature will include Google Scholar, OpenGrey, RIAN, EThOS, ProQuest, WorldCat, Networked Digital Library of Theses and Dissertations, Open Access Theses and Dissertations, and public health organisation websites. All search results will be reviewed for eligibility, except in the case of Google Scholar where the first 200 citations from this search engine will be screened. This is based on research which shows that optimal searches in Google Scholar, for purposes of systematic reviews, should include a minimum of 200 references sorted by relevance [46, 47]. In addition, the lead or corresponding authors of all included studies will be contacted (via email with two attempts) to identify ongoing or unpublished research studies relevant to this review. Moreover, the reference lists of included studies will be searched for relevant studies and reference chaining of all included studies will be conducted. To ensure that the search strategy is implemented in a systematic manner, a memoing process will be used to record the working notes of the lead review author (CK) when conducting the iterative search process as well as documenting the protocol-driven search strategy [48].

**Table 1:** PubMed search strategy

Search number	Search string
#1	restaurant* [tiab] OR cafeteria* [tiab] OR canteen* [tiab] OR fast food [tiab] OR vending machine* [tiab] OR menu* [tiab] OR food service [mh] OR food service* [tiab]
#2	food labeling [mh] OR label* [tiab] OR post* [tiab]
#3	calorie* [tiab] OR kilojoule* [tiab] OR energy [tiab] OR nutri* [tiab]
#4	#1 AND #2 AND #3

*mh* MeSH headings; *tiab* title/abstract

### Study selection

The lead review author (CK) will upload the search results and remove duplicates using EndNote X7 (Clarivate Analytics). Search results will then be imported into Covidence, an online systematic review software, to conduct relevance screening, data extraction and quality assessment. Three review authors (CK, CH and another review author to be confirmed) will independently screen study titles and abstracts to decide whether the full-text manuscript should be retrieved. Each study will be classified as either (a) potentially meeting the eligibility criteria or (b) not meeting the eligibility criteria for inclusion. Studies potentially meeting the inclusion criteria will be obtained in full-text format. Three review authors (CK, CKelly and FG) will independently complete the full-text screening process. Another review author (IJP) will be consulted if discrepancies during study selection are not resolved by consensus. A flow diagram will be used to report the study selection process as recommended by the PRISMA guidelines [44].

### Data extraction

Two review authors (CK and JMS) will independently extract data from the included studies, in an unblinded standardised manner, using a data extraction form. To assess validity and reliability of the data extraction form, a pilot will be undertaken on a subset of included studies by the two independent reviewers. Afterward, the two reviewers will compare the data extraction and modifications will be made to the data extraction form where required. The data extraction form will consist of the following sections: (a) key study information, (b) a coding manual with definitions for each of the 39 CFIR constructs, (c) new themes for evidence and (d) the quality assessment criteria. Key study information will include study title, name of the first author, year of publication, time of data collection (i.e. year), country of study, study type (qualitative, quantitative and mixed methods studies), intervention type (quantitative (e.g. calories, sodium, fat) or qualitative (e.g. traffic light labelling, healthy-food symbols) menu labelling format), menu labelling scheme (voluntary or mandatory participation), setting (fast food, restaurant, coffee shop etc.) and sample (restaurant manager, policy-maker etc.). Relevance to the workplace and healthcare setting will also be extracted, as per the secondary outcomes of this review. Data on barriers and facilitators will be extracted from the results and discussion sections of the included studies. This will include (i) verbatim quotations from research participants; (ii) excerpts, quotations or entire passages from studies using documentary analysis; (iii) narrative descriptive summaries of results; and (iv) statistical analyses from surveys and questionnaires. The rationale for extracting data from the results and discussion sections is based on findings that raw data from qualitative research may be presented in both sections [49–51]. If any discrepancies arise during the data extraction process, these will be resolved by consensus and discussion with a third reviewer (IJP) if required.

### Quality assessment

The Mixed Methods Appraisal Tool (MMAT) [52] will be used to appraise the qualitative, quantitative and mixed methods studies for this review. The MMAT has established content validity and has been piloted across all methodologies [53, 54]. For each study type, an overall quality score will be calculated. In the case of poor reporting of qualitative research, study authors will be contacted for additional information. This is considered standard practice when reviewing qualitative studies [55, 56]. Two independent reviewers (CK and JMS) will perform the critical appraisal of all included studies. To facilitate comparison of appraisal scores, both reviewers will record the rationale for study scores and the relevant location in the full-text articles. A third reviewer (IJP) will be consulted in the case of disagreement without reaching consensus. No study will be excluded based on quality assessment, as they may still offer valuable insight [57].

### Data synthesis

The “best fit” framework synthesis method [42] will be led by CK and CKelly, with input from the review team. The first step involves coding the data extracted from studies against the CFIR. To ensure the accuracy of this coding process, the data extraction form will contain a coding manual for the CFIR, which includes pre-established definitions for existing constructs [45]. Then, secondary thematic analysis will be undertaken of information that does not fit within the CFIR [58]. Concepts from both the deductive and inductive analysis will then be clustered and synthesised into a final set of themes representing the whole dataset. Both reviewers (CK and CKelly) will then identify the relationships between themes based on relevant theories and evidence from the primary research studies. The overall process will assist in the construction of a new evidence-based conceptual model regarding the implementation of menu labelling interventions to support healthy food choices. The new model will then be critically considered by all members of the review team until consensus is reached. Finally, findings from studies relating to the workplace and healthcare setting will be compared with the synthesis to identify any potential differences.

### Testing the synthesis

Following the construction of a new conceptual model, CK will then assess the potential for bias and conduct a sensitivity analysis. As per the ‘best fit’ framework synthesis approach [42], this will involve exploring any differences between the CFIR and the new conceptual model so as to provide explanations for the absence of particular themes and the addition of new themes. This process will help to understand and contextualise the findings of the new model in relation to the a priori framework, as well as assessing for publication bias. For example, it will explore if the absence of CFIR constructs in the new model is related to understandable differences in the intervention and/or setting or if the absence needs to be explored further by revisiting the literature. Furthermore, in the absence of multiple cases of dissonance, purposive efforts will be made to identify negative cases within the evidence base. This will involve searching the review findings for evidence that serve as examples that do not fit emergent patterns (i.e. that oppose or limit the initial results in some way) [59]. Moreover, a sensitivity analysis will be conducted in order to determine if the synthesis is sensitive to the following: study design, quality assessment, time of data collection (i.e. year), intervention type (quantitative or qualitative menu labelling format), menu labelling scheme (voluntary or mandatory participation), setting (e.g. fast food, restaurant, coffee shop), sample (e.g. restaurant manager, caterer, policy-maker) and location (e.g. Europe, America, Australia). As barriers and facilitators to implementation are highly context-dependent, the sensitivity analysis will be an important step in assessing if the synthesis is sensitive to contextual factors such as intervention type, menu labelling scheme and time of data collection. Overall, the bias and sensitivity review process will help ensure reflexivity, rigour and quality.

## Discussion

Implementation of menu labelling interventions is a complex process that needs to be fully explored in order to increase understanding of and support for successful implementation. To date, there has been limited research on the implementation of such interventions and no systematic attempt to synthesise the evidence-base. This mixed methods systematic review aims to address this gap by providing insights into what enables or hinders stakeholders in the implementation of menu labelling interventions. Incorporating different types of evidence, both from qualitative and quantitative research, will strengthen the review findings and make them more relevant to policy and practice [60]. This mixed methods review will allow for a more complete and comprehensive understanding of the barriers and facilitators to implementing menu labelling interventions than either quantitative or qualitative approaches alone.

In this review, the CFIR has been chosen as the a priori framework because of its broad applicability to the area under review. The CFIR incorporates constructs from a range of implementation theories and therefore is more likely to accommodate a significant amount of data [45]. Moreover, using a meta-framework such as the CFIR makes it less likely that the review team will over-look important themes. Likewise, the use of clear consensual definitions for each of the 39 CFIR constructs will enhance the reliability of coding and also strengthen the rigour of the synthesis. The combination of the deductive coding (using the CFIR to guide identification) and inductive analysis (allowing new themes to emerge) is a strength of the ‘best fit’ framework approach [45].

The review will incorporate a number of strategies to help minimise the effects of meta-bias and to improve overall validity and reliability [61, 62]. Firstly, the review will include unpublished and grey literature to reduce the risk of publication bias. Similarly, no language restrictions will be applied, thereby reducing the potential for language bias. Furthermore, the risk of selection bias will be minimised with two independent reviewers performing study selection, data extraction and quality appraisal. Secondly, the PRISMA guidelines will be followed when conducting and reporting the systematic review [44]. As with any knowledge synthesis, the documentation of the literature search process will be imperative to ensure transparent and reproducible search methods. Thirdly, as all studies meeting the inclusion criteria will be included in this review, necessary steps will be taken to address concerns relating to risk of bias and lack of rigour (e.g. quality assessment, sensitivity analysis). Finally, where deviations from this protocol occur, this will be justified and discussed in the systematic review upon publication.

We anticipate that the findings from this review will be highly relevant to a wide array of stakeholders including food service business staff and management, policy-makers, guideline developers and researchers. Findings will provide insight on the barriers and facilitators that hinder or enable implementation of menu labelling interventions; therefore, advising policy-makers and other stakeholders involved in the roll-out of such interventions, and informing their future development and implementation. The results of this mixed methods systematic review will be widely disseminated through publication in a peer-reviewed, open-access journal, presentations at conferences, seminars and workshops and via social media.

## Additional file

Additional file 1: PRISMA-P (Preferred Reporting Items for Systematic review and Meta-Analysis Protocols) 2015 checklist: recommended items to address in a systematic review protocol. This file provides a completed PRISMA-P 2015 checklist. (DOC 85 kb)

## Abbreviations

CFIR: Consolidated Framework for Implementation Research
FSAI: Food Safety Authority of Ireland
MMAT: Mixed Methods Appraisal Tool
PRISMA: Preferred Reporting Items for Systematic Reviews and Meta-Analyses
ROI: Republic of Ireland
UK: United Kingdom
USA: United States of America
