# Supplementary material for: Barriers and facilitators to implementation of menu labelling interventions to support healthy food choices: a mixed methods systematic review protocol
Source: Syst Rev. 2018 Jun 23;7:88. doi: 10.1186/s13643-018-0752-3 (PMC6015453; doi:10.1186/s13643-018-0752-3)
Supplement: Supplementary file 1 — PRISMA-P (Preferred Reporting Items for Systematic review and Meta-Analysis Protocols) 2015 checklist: recommended items to address in a systematic review protocol. This file provides a completed PRISMA-P 2015 checklist. (DOC 85 kb) [file 13643_2018_752_MOESM1_ESM.doc]

**Additional file 1: PRISMA-P (Preferred Reporting Items for Systematic review and Meta-Analysis Protocols) 2015 checklist: recommended items to address in a systematic review protocol**

| Section and topic | Item no. | Checklist item |
| --- | --- | --- |
| ADMINISTRATIVE INFORMATION | | |
| Title: |  |  |
| Identification | 1a | Identify the report as a protocol of a systematic review  **See page 1, lines 1-2.** |
| Update | 1b | If the protocol is for an update of a previous systematic review, identify as such  **Not applicable.** |
| Registration | 2 | If registered, provide the name of the registry (such as PROSPERO) and registration number  **See page 3, line 63.** |
| Authors: |  |  |
| Contact | 3a | Provide name, institutional affiliation, e-mail address of all protocol authors; provide physical mailing address of corresponding author  **See page 1, lines 4-28.** |
| Contributions | 3b | Describe contributions of protocol authors and identify the guarantor of the review  **See page 21, lines 478-482.** |
| Amendments | 4 | If the protocol represents an amendment of a previously completed or published protocol, identify as such and list changes; otherwise, state plan for documenting important protocol amendments  **See page 19, lines 434-435.** |
| Support: |  |  |
| Sources | 5a | Indicate sources of financial or other support for the review  **See page 20, lines 473-474.** |
| Sponsor | 5b | Provide name for the review funder and/or sponsor  **See page 20, lines 473-474.** |
| Role of sponsor or funder | 5c | Describe roles of funder(s), sponsor(s), and/or institution(s), if any, in developing the protocol  **Not applicable.** |
| INTRODUCTION | | |
| Rationale | 6 | Describe the rationale for the review in the context of what is already known  **See pages 4-8, lines 77-189.** |
| Objectives | 7 | Provide an explicit statement of the question(s) the review will address with reference to participants, interventions, comparators, and outcomes (PICO)  **See page 8, lines 179-181.** |
| METHODS | | |
| Eligibility criteria | 8 | Specify the study characteristics (such as PICO, study design, setting, time frame) and report characteristics (such as years considered, language, publication status) to be used as criteria for eligibility for the review  **See pages 9-12, lines 217-275.** |
| Information sources | 9 | Describe all intended information sources (such as electronic databases, contact with study authors, trial registers or other grey literature sources) with planned dates of coverage  **See pages 12-13, lines 284-300.** |
| Search strategy | 10 | Present draft of search strategy to be used for at least one electronic database, including planned limits, such that it could be repeated  **See page 13, lines 305-306.** |
| Study records: |  |  |
| Data management | 11a | Describe the mechanism(s) that will be used to manage records and data throughout the review  **See page 14, lines 311-314.** |
| Selection process | 11b | State the process that will be used for selecting studies (such as two independent reviewers) through each phase of the review (that is, screening, eligibility and inclusion in meta-analysis)  **See pages 14, lines 314-321.** |
| Data collection process | 11c | Describe planned method of extracting data from reports (such as piloting forms, done independently, in duplicate), any processes for obtaining and confirming data from investigators  **See page 14-15, lines 325-347.** |
| Data items | 12 | List and define all variables for which data will be sought (such as PICO items, funding sources), any pre-planned data assumptions and simplifications  **See page 14-15, lines 330-345.** |
| Outcomes and prioritization | 13 | List and define all outcomes for which data will be sought, including prioritization of main and additional outcomes, with rationale  **See page 11, lines 246-257.** |
| Risk of bias in individual studies | 14 | Describe anticipated methods for assessing risk of bias of individual studies, including whether this will be done at the outcome or study level, or both; state how this information will be used in data synthesis  **See page 16-17, pages 379-400.** |
| Data synthesis | 15a | Describe criteria under which study data will be quantitatively synthesised  **Not applicable.** |
|  | 15b | If data are appropriate for quantitative synthesis, describe planned summary measures, methods of handling data and methods of combining data from studies, including any planned exploration of consistency (such as I^2^, Kendall’s τ)  **Not applicable.** |
|  | 15c | Describe any proposed additional analyses (such as sensitivity or subgroup analyses, meta-regression)  **See page 16-17, lines 379-400.** |
|  | 15d | If quantitative synthesis is not appropriate, describe the type of summary planned  **See pages 15-16, lines 363-376.** |
| Meta-bias(es) | 16 | Specify any planned assessment of meta-bias(es) (such as publication bias across studies, selective reporting within studies).  **Not applicable.** |
| Confidence in cumulative evidence | 17 | Describe how the strength of the body of evidence will be assessed (such as GRADE)  **Not applicable.** |
